# Design and Application of a Novel Silicone Nasal Implant

**DOI:** 10.1093/asjof/ojad040

**Published:** 2023-05-04

**Authors:** Jianjun Zhang

## Abstract

**Background:**

Traditional silicone implants used in augmentation rhinoplasty lead to postoperative complications.

**Objectives:**

To introduce a novel silicone implant designed to reduce postoperative complications.

**Methods:**

The author designed a novel modification for the traditional silicone nasal implant, which has a particle surface, vertical and horizontal grooves, and a special vertical board to support the nasal tip. A total of 114 consecutive clinical cases were retrospectively reviewed from September 2016 to November 2022, with a minimum of 36 months and an average 51 months of follow-up. All patients underwent augmentation rhinoplasty using this novel implant, with 97 (85.09%) patients using only the silicone and 17 (14.91%) the silicone implant with conchal cartilage. Surgical complications such as sliding down, redness, extrusion, deviation, translucency, capsular contracture, or infection were recorded.

**Results:**

The median patient age was 28 (range, 18-55) years, with 109 female and 5 male patients. Among the 114 cases, 46 (40.35%) involved primary surgery and 68 (59.65%) involved revisional surgery. The overall complication rate was 4.39%, and 0.88% of the patients had slight redness, 0.88% had intermittent pain, and 2.63% had infections. No other complication was observed, and all complications occurred in revisional surgeries. A total of 109 patients (95.61%) showed satisfying results without any postoperative complication. None of the patients with primary surgery reported postoperative complications.

**Conclusions:**

The novel silicone nasal implant can effectively reduce the rate of postoperative complications. Therefore, augmentation rhinoplasty using this implant enables a more natural-looking outcome.

**Level of Evidence: 3:**

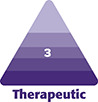

Augmentation rhinoplasty with nasal implants is one of the most commonly performed aesthetic surgical procedures in Asian countries. During augmentation rhinoplasty with silicone nasal implants, the implant is carved and placed onto the subcutaneous layer of the nose. This modifies the curve of the nasal dorsum and increases the nasal tip projection to meet aesthetic standards. The silicone nasal implant is favored, given its good biocompatibility, easy tailoring, and affordability. However, postoperative complications such as postoperative implant deviation, translucency, extrusion, and capsular contracture still occur.^1^

Modification and optimization of the structure of the implant could effectively reduce the risk of postoperative complications. In this study, a total of 114 clinical patients treated from September 2016 to December 2019 were reviewed. All patients underwent augmentation rhinoplasty using a novel silicone nasal implant with a modified and optimized structure. In this study, we aimed to introduce a novel silicone implant designed to reduce postoperative complications.

## METHODS

### An Ideal Nasal Implant Design

In reality, all prostheses for rhinoplasty have some limitations. Therefore, surgeons are continuously trying to determine a better surgical plan or nasal implant to improve operative outcomes and reduce surgical complications. From the author's perspective, the ideal nasal implant design should meet the following requirements:

1. It should have a small volume but a large surface area to reduce the risk of capsular contracture.
2. It should have a rough surface to increase friction and reduce sliding.
3. The tip of the implant should be able to swing freely without moving the nasal bridge of the implant.
4. The postoperative nose should be soft to the touch at both the tip and the dorsum.
5. The resulting nose front should provide a lifting force toward the tip.
6. It should produce a smooth transition at the nasofrontal area.
7. The implant shape should have a physiological curve that matches the natural shape of the nose.

### Implant Design

**Figure 1.**
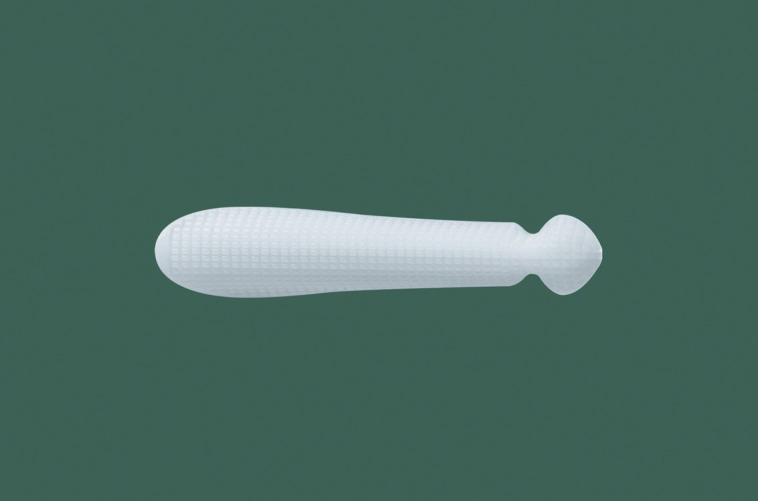
Top view of the Yusha implant (Model T40-650, 6 mm thickness and 50 mm length; Guangzhou Wanhe Plastic Materials Co., Ltd., Guangzhou, China).

**Figure 2.**
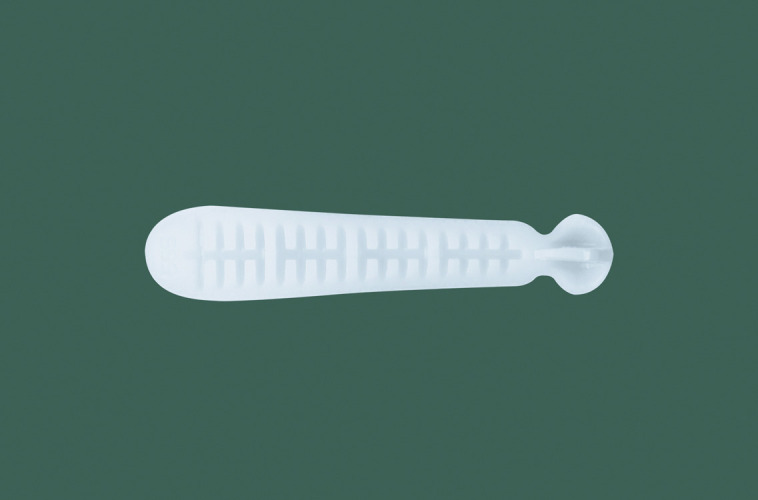
Bottom view of the Yusha implant (Model T40-650, 6 mm thickness and 50 mm length; Guangzhou Wanhe Plastic Materials Co., Ltd.).

**Figure 3.**
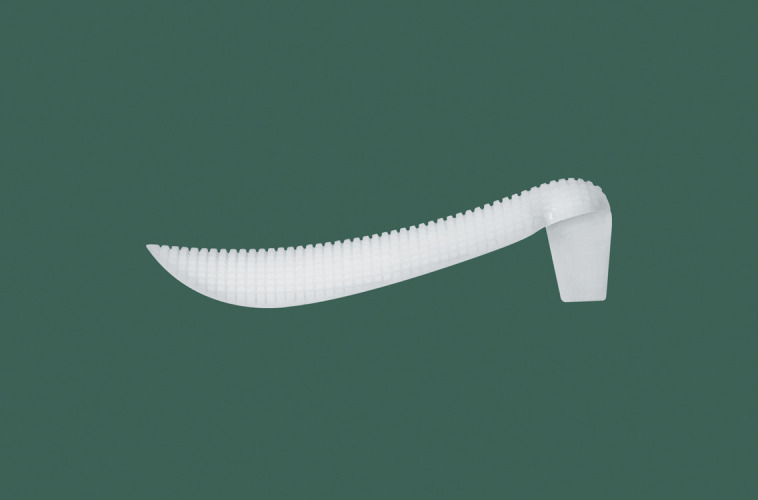
Side view of the Yusha implant (Model T40-650, 6 mm thickness and 50 mm length; Guangzhou Wanhe Plastic Materials Co., Ltd.).

To design this novel implant, the author made various changes to the physical structure of the traditional silicone nasal implant. The purpose behind all these changes was to produce a postoperative result that imitates the natural human nose in terms of outside appearance, function, and tactile sensation. Following this, the revised implant had the following characteristics:

1. The dorsal side of the implant is a convex particle surface with a convexity of 0.5 mm; it is composed of countless granular projections with a length of 0.8 mm and a width of 0.4 mm spaced 0.4 mm from each other. The convex particle surface can form a diffuse reflection, which reduces the risk of translucency.
2. The main vertical ventral groove is accompanied by 12 to 16 horizontal grooves on both sides. The groove depth is 2 to 3 mm. Both vertical and horizontal grooves provide higher friction and therefore prevent the implant from sliding down.
3. A “mantis neck,” which is a narrowed neck, is located at the junction of the tip and body of the implant. This design imitates the human nose tip structure where the alar cartilages connect to the nasal bone.
4. The implant is intentionally hollowed, which enables a soft nose tip.
5. A hole allows soft tissue and vessels to pass through the implant and improves the blood circulation of the skin on the back of the nose. This reduces the whitening of the back of the nose and fixes the implant in place.
6. The columella (later referred to as “the vertical board”) is connected to the tail of the nasal septum. The thin, vertical board design can provide enough support to the nasal tip without increasing the width of the columella.
7. The design drawings of the new silicone nasal implant are shown in Figures 1–3. The implant was named the Yusha nasal implant (Guangzhou Wanhe Plastic Materials Co., Ltd., Guangzhou, China).

The Yusha implant has 20 different models in total, including models with thicknesses of 3, 4, 5, 6, and 7 mm and lengths of 45, 50, 55, and 60 mm. It has been certified by the China Food and Drug Administration under the registration certificate number 20143460494 on March 21, 2014.

### Preoperative Consultation

Preoperative consultation was focused on the characteristics of patients, including but not limited to nasal figure, operational history, and physical condition. Before an operation, the patient's face contour and nose conditions were inspected. Then, the nose length and level of nasal deformity were measured. A surgical plan and choice of prosthetic model were discussed with the patient based on their subjective desires. Surgery was performed only after obtaining the patient's written informed consent. The novel silicone nasal implant was used for the rhinoplasty procedure.

Surgery was not performed if the patient had a false perception regarding the safety of the silicone implant, disagreement with the implant design, or unrealistic expectations regarding the outcomes of the surgery (expecting a significant difference in the postoperative nose tip or a nose back height that is not based on the patient's original nose shape). Meanwhile, an examination was performed to identify any ulceration or erosion on the patient's nose. The patient was also asked to provide medical history, including surgical history and any allergy to medication. Routine blood tests for liver function, kidney function, and blood sugar were also performed, as appropriate.

### Surgical Method

The hair inside the nostril was trimmed before surgery, and the secretions in the nasal cavity were cleaned. For primary surgery, the author usually used local anesthesia consisting of 1% lidocaine mixed with 1:100 000 epinephrine. For patients who could not tolerate the pain of local anesthesia injection, intravenous anesthesia was used. For revisional surgery that is longer than 2 h, general anesthesia with tracheal intubation was used. Then, 0.3% iodophor was used twice to disinfect the skin on the face, while being careful to protect the cornea and prevent the disinfectant from entering the eyes by mistake. Next, 0.3% iodophor was used twice to disinfect the nasal vestibule and then set up sterile surgical drapes. Both local and general anesthesia needed extra 2% lidocaine injected at the surgical area.

The surgeon first made a wing-shaped open incision at the columella. The incision was extended along the nostril edge of the nasal columella to the right and left alar. Careful attention was required to ensure that the incision was located 2 mm away from the bilateral alar rims. The skin of the nasal columella close to the surface of the alar cartilage was lifted to expose a part of the bilateral alar cartilage. A subperiosteal dissection was then performed above the surface of the nasal bone and under the deep fascia. The upper dissection was limited slightly below the junction point of the 2 eyebrows according to a preplanned mark. The dissection led to the formation of an adequate prosthesis pocket where the implant could be placed. In revisional surgeries, removal of a previous implant or residual cartilage was needed. Silicone nasal implants were usually removed easily, but polytetrafluoroethylene (ePTFE) material was very hard to remove. The ePTFE was carefully dissected from the surrounding tissue, while avoiding damage to the skin around the nasal dorsum.

**Figure 4.**
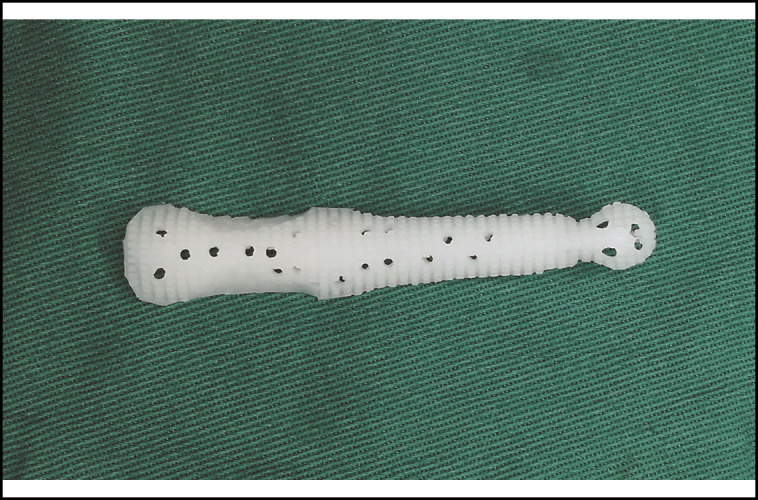
Top view of a carved Yusha implant (Guangzhou Wanhe Plastic Materials Co., Ltd.).

**Figure 5.**
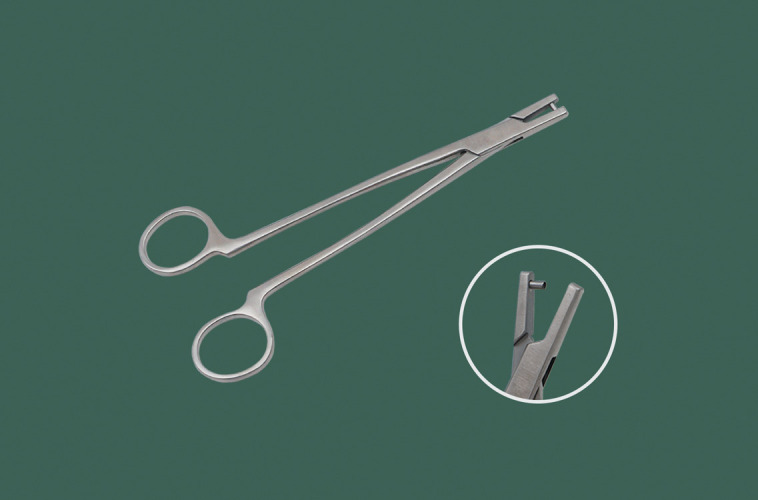
Hole puncher.

**Figure 6.**
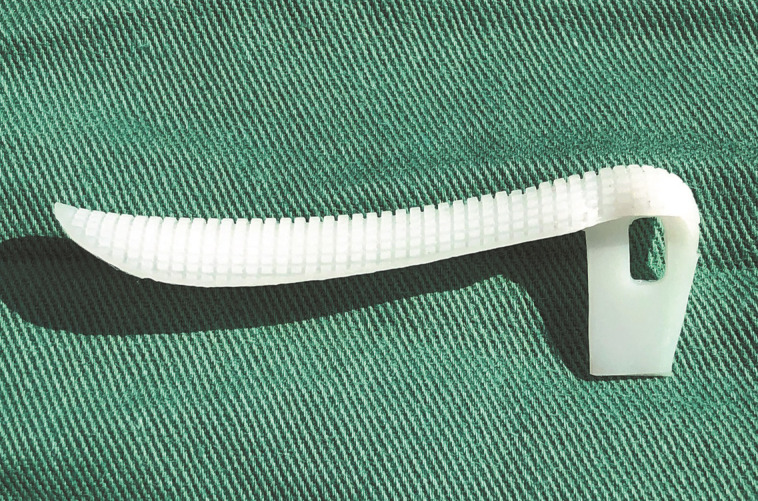
Side view of a carved Yusha implant (Guangzhou Wanhe Plastic Materials Co., Ltd.).

In revisional surgeries with hyaluronic acid injection history, the prosthesis pocket was rinsed thoroughly and cleaned with a spatula to ensure no hyaluronic acid was left inside. The implant could be carved either before sedation or after the pocket space was done. The engraving of the implant was performed in a manner that resulted in the following:

- The transition between the end of the implant (which connects to the nasofrontal line) and the forehead was smooth and wide.
- The horizontal canthus part of the implant was narrowed moderately.
- The position of the implant corresponded with the tendon stone area, maintaining enough width and thickness.
- The lower half of the implant (near nasal tip) was narrowed to a width of ∼6 mm (Figure 4).
- Around 15 to 20 holes were punched in the implant, each hole being 1.5 mm wide, throughout the body (a puncher was also designed for this purpose) (Figure 5).
- The upper part of the vertical board of the columella was partially cut out to make the nose tip feel softer (Figure 6).

The carved Yusha nasal implant was inserted into the surgical pocket and adjusted to a central position. A deep cavity was then dissected into the caudal end of the nasal septum for the insertion of the vertical board of the prosthesis. The vertical board was inserted into the cavity as the medial foot of the bilateral alar cartilage. The bilateral fornix of the alar cartilage was sutured to fix the prosthesis in place.

The patient, if under local anesthesia, was allowed to observe the postoperative result using a mirror to gauge the levels of satisfaction. Then, the incisions were closed after the operation if blood oozing was not detected. A thermoplastic plate was applied to fix a compress bandage for 72 h. The suture was removed on the eighth postoperative day.

### Evaluation Standard

**Figure 7.**
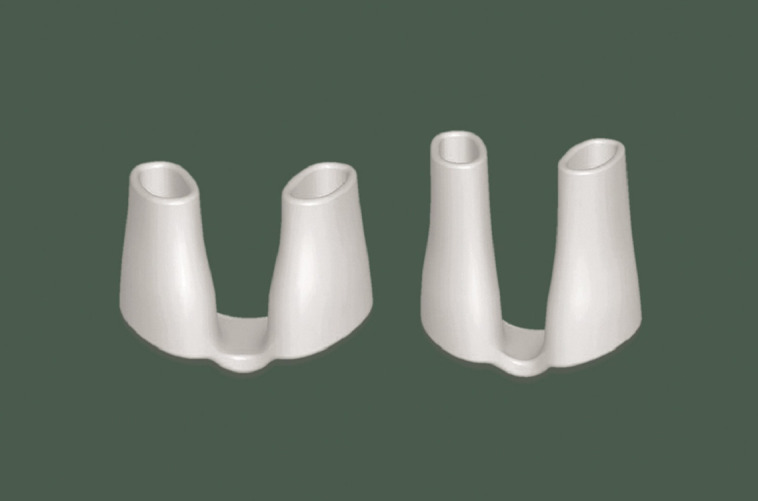
Silicone nasal dilator.

Postoperative results were evaluated based on 3 aspects: surgical complication, physiological function, and aesthetic integrity. To gauge patient satisfaction, these aspects were assessed in the following 3 critical postsurgery periods:

1. In the first stage of wound healing, which usually occurs within 2 weeks after surgery, a satisfactory surgical outcome should involve no hematoma or infection. Regarding nasal function, in the first healing stage, it is normal to have some difficulty in breathing; therefore, patients were advised to use a silicone nasal dilator to improve breathing and nasal airflow (Figure 7). This device can also help in shaping the postoperative nose and fixing the nasal columella. In this stage, swelling is common, especially in revisional surgery, so less attention is paid to the appearance of the nose. Asymmetry caused by uneven swelling is acceptable.
2. From 2 weeks to 3 months after surgery, the wound should complete the initial phrase of healing. We observed for the occurrence of any infection, redness, deviation, sliding, or implant exposure. If any of the above complication occurs, patient satisfaction will drop significantly. At this stage, nose function should regain normalcy.
3. After 3 months, the nose should regain complete normalcy with no surgical complications. There should be no tension in the wound area. Appearance is the most important factor that dictates patient satisfaction. The implant should be well integrated into the nasal structure and should not be visible from the outside nor have any deviation.

In a dynamic situation, the nose back should stay fixed while the nose front moves. This is tested by pinching the back of the nose with the thumb and index finger and moving it to the left and right. If the implant stays steady, it is judged to be satisfactory. The nose tip should not have too much tension. The surgeon can assess this by comparing the feel of their own nose with that of the patient's postsurgical nose. A similar feeling is a satisfactory result.

Functionally, the patient should be able to breathe and sneeze as normal, and the nostril should fit at least the little finger.

## RESULTS

### Clinical Data

A total of 114 cases were retrospectively reviewed (Table 1). The patients were all females, with age ranging from 18 to 55 (mean, 28.27) years. Forty-six patients did not have a history of rhinoplasty; 68 were unsatisfied with the results of implant rhinoplasty performed elsewhere, among which 32 were unsatisfied with the results of normal silicone implant rhinoplasty, 25 were unsatisfied with the results of hyaluronic acid injection or polydioxanone (PDO) threads rhinoplasty, 7 were unsatisfied with the results of expanded ePTFE implant rhinoplasty, and 4 were unsatisfied with the results of rhinoplasty with both ePTFE and costal cartilage.

**Table 1.** Statistics of the Novel Nasal Implant Postoperative Patients’ Complications

Preoperative condition		No. of cases	No. of satisfied patients	Unsatisfied result	Percentage of total cases
1. Primary cases (never underwent rhinoplasty before)		46	46	0	0.00
2. Revision cases (have undergone rhinoplasty previously, but not satisfied)	2.1 Injected hyaluronic acid/polydioxanone (PDO)	25	24	1	0.88
	2.2 Silicone implant	32	31	1	0.88
	2.3 polytetrafluoroethylene (ePTFE) implants solely or ePTFE implants with other materials	11	8	3	2.63
	Subtotal	68	63	5	4.39
Total		114	109	5	4.39

The follow-up period ranged from 36 months to 75 months. Out of the 114 patients, 109 had satisfied outcomes and did not experience any postoperative complications, 1 had slight redness, 1 experienced periodic pain, and 3 developed infections. All complications occurred in revisional surgery. The total unsatisfied outcome rate due to complications was 4.39%, and the infection rate was 2.63%. Patients who desired reoperations due to aesthetic dissatisfaction were not included in the complication rate above.

### Cases of Unsatisfying Results

#### Redness

A 23-year-old female patient underwent revisional surgery after hyaluronic acid injection. The patient presented slight redness after surgery and, therefore, the implant was withdrawn.

#### Periodic Pain

A 30-year-old female patient who underwent revisional surgery for traditional L-shape silicone implant had periodic pain after surgery; therefore, the implant was removed.

#### Infection

A 22-year-old female patient had infection from a small remnant of the ePTFE implant from a previous surgery but healed after cleaning of that remnant. Two months after the removal of the ePTFE part, the patient asked for a reoperation using the Yusha implant. However, a new infection occurred after the reoperation, leading to the removal of the new implant.

A 32-year-old female patient had revisional rhinoplasty for an ePTFE implant with allogeneic bone graft and ear cartilage. This patient developed infection after surgery and, therefore, had to remove the implant.

A 25-year-old female patient had revisional surgery for an ePTFE implant and costal cartilage. The patient had infection after surgery and therefore had the implant removed.

### Cases With Satisfying Results

#### Case 1: A 21-Year-Old Female Patient

**Figure 8.**
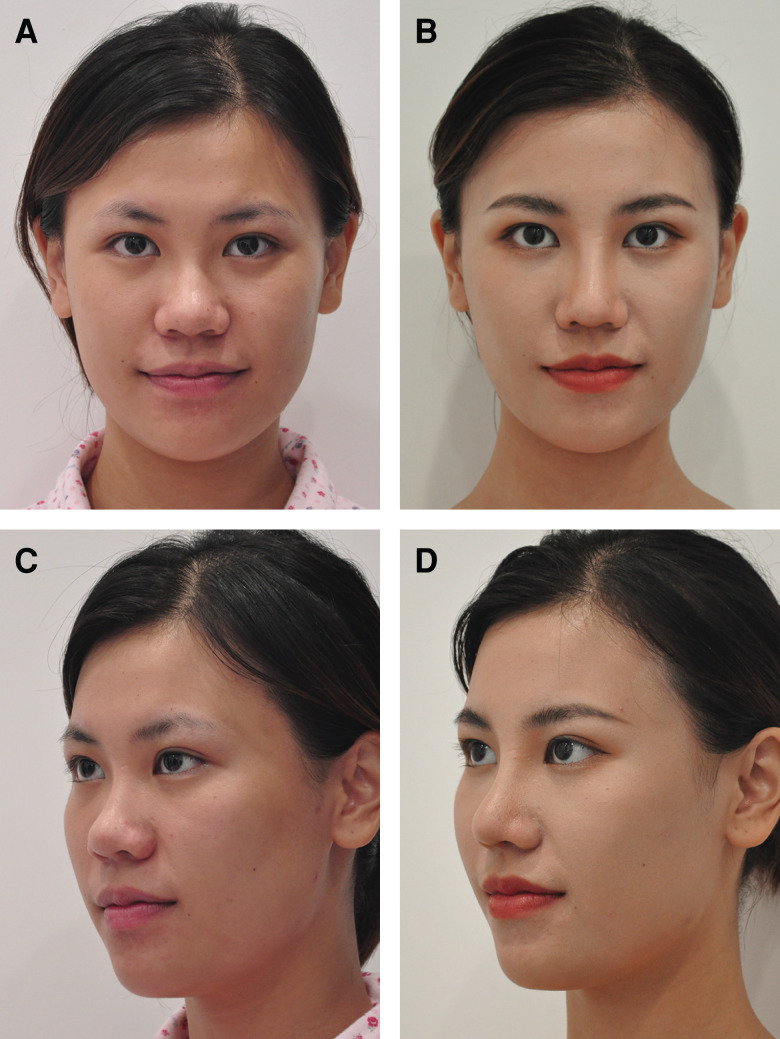
A 22-year-old female patient who underwent revisional surgery after hyaluronic acid injection. Preoperative and 35-month postoperative (A, B) frontal view and (C, D) 3-quarter view photographs are shown.

The patient had a history of hyaluronic acid injection, and the hypertrophy of her nasal tip was obvious. During the revisional surgery, the injected hyaluronic acid was removed, and the cavity was rinsed. The nasal tip was reshaped, and a rhinoplasty was subsequently performed using the Yusha nasal implant (Video). The patient was satisfied with the results of the surgery during a follow-up consultation 5 months after the procedure (Figure 8).

#### Case 2: A 33-Year-Old Female Patient

**Figure 9.**
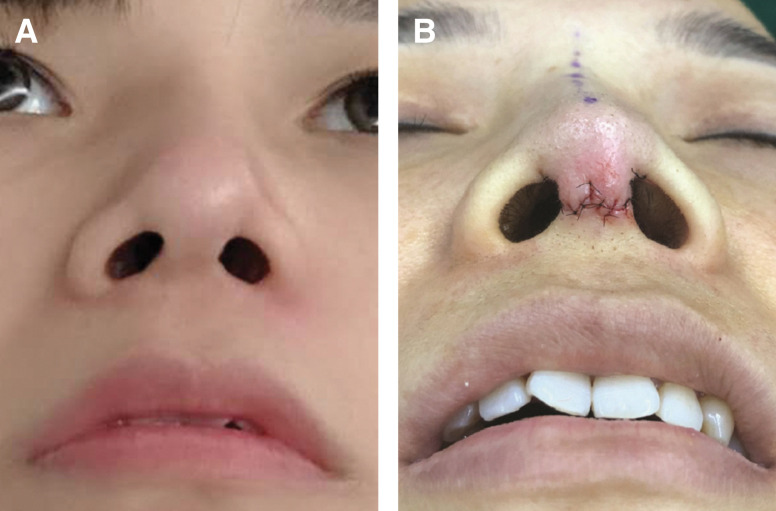
A 33-year-old female patient who underwent revisional surgery after a traditional L-shape silicone implant. (A) Preoperative and (B) immediate postoperative top view photographs are shown.

A deviation of the nasal dorsum and columella was observed in a patient 1 year after rhinoplasty involving the conventional silicone implant. The conventional implant was replaced with the Yusha nasal implant. An adjustment was made to the surgical cavity (Figure 9). The patient was satisfied with the results 6 months after the surgery during a follow-up consultation.

#### Case 3: An 18-Year-Old Male Patient

This patient underwent hyaluronic acid injection 12 months before preoperative consultation. The nose presented a wide back with insufficient tip projection.

During the revisional surgery, the injected hyaluronic acid was removed and the Yusha implant was used. The patient was satisfied with the results 25 months after the surgery during a follow-up consultation.

## DISCUSSION

In recent years, various rhinoplasty materials have emerged in a seemingly endless stream, from hyaluronic acid injection and ePTFE, to allogeneic bone and costal cartilage. These materials all have their own advantages and disadvantages.

Hyaluronic acid has an increased demand in the Asian market because of its convenience and short recovery period. However, vascular complications have always been a serious concern of this procedure. Intravascular injection or the compressive effect of the filler on local vessels might lead to extensive skin necrosis.^1,2^

Furthermore, according to personal experience, the long-term appearance of rhinoplasty with hyaluronic acid injection is usually not satisfactory. The nasal dorsum usually starts to widen 1 month after injection. The gel may cause embolism in other parts of the body if it enters the bloodstream. In particular, embolism of the ophthalmic vessels near the site of the injection will cause blindness.^2,3^ This surgical method should not be recommended.

**Figure 10.**
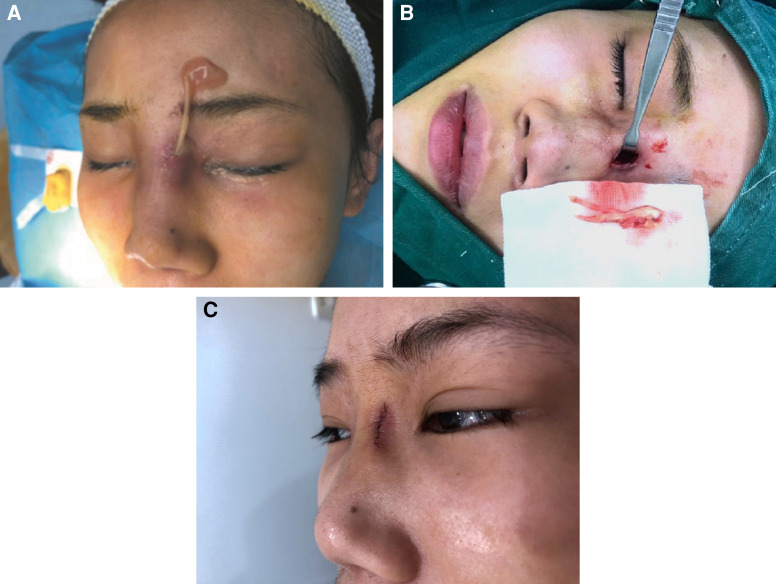
A 27-year-old female patient who underwent revisional surgery after ePTFE rhinoplasty and developed postoperative infection because a tiny piece of ePTFE was left inside. (A, B) Drainage was performed 20 days after surgery and (C) it removed the left-out piece of ePTFE. ePTFE, polytetrafluoroethylene.

ePTFE is a porous material with a pore size ranging from 10 to 30 μm.^4,5^ The advantage of ePTFE is that it allows ingrowth of the tissue, which might prevent migration of the prosthesis, while minimizing contracture due to higher stability.^6,7^ However, due to its porous structure, this ingrowth can complicate removal.^5,8^ Lack of capsule formation also means that implants are closer to the skin and therefore more visible.^6^ In addition, in 1993, Maas et al studies on animal models showed that when ePTFE was placed inside the host, it would cause minor inflammation that gradually diminished and stabilized.^4,9^ However, because microbes can enter the micropores, but macrophages with a diameter of more than 50 μm cannot, human macrophages cannot target microbes, which leads to frequent postoperative infections.^4,9^ Winkler et al reported that in rhinoplasty surgeries in which ePTFE was used alone, the infection rate was 5.3%.^10^ This study included a patient who did not heal for a long time, even after her ePTFE implant was taken out. She had a large abscess that was difficult to cure, despite a drainage procedure, taking more than a month to heal. Surgical debridement had to be performed to check for the source of the infection, revealing that a tiny piece of ePTFE left behind in the patient's nose was causing the infection. The incision healed 1 week after debridement (Figure 10).

Because ePTFE is manufactured using molding, stretching, and superimposing, it is available only in sheets or blocks. Surgeons must carve the ePTFE blocks from scratch to obtain the desired shape during the operation, which requires a high level of carving skill and prolonged OR time. Therefore, ePTFE is difficult to configure in moderate augmentation with variable curves.^7^ Moreover, ePTFE does not have the rigidity required to shape the nasal tip and columella.^11^

Some surgeons prefer using costal cartilage or septal cartilage when constructing the tip structure to increase the tip projection. Cartilages are biocompatible, with near-zero infection and resorption rates.^12^ Costal cartilage provides sufficient bulk, but donor site pain, scarring, and risk of pneumothorax still remain as drawbacks. This not only increases trauma during an operation but also creates a sharp, hard, and artificial look on the nasal tip. This technique significantly increases skin tension, reduces postoperative nose softness, narrows the nostrils, and produces an abnormally widened columella.^13^ Long-term follow-up also shows a higher rate of revisional operations.

Rhinoplasty with septal cartilage is also a common procedure. The nasal septal cartilage plays an important role in supporting the nasal dorsum and tip and separating the 2 nasal cavities. The nasal septal cartilage in Asian patients is inherently and insufficiently developed, especially in patients who need augmentation rhinoplasty.^14,15^ Therefore, most of the time, the harvested septal cartilage cannot provide sufficient material to achieve an ideal tip projection. Rhinoplasty with implants can reduce the risk of injury to the nose and other parts of the human body when compared with rhinoplasty involving harvesting cartilage, because removal of cartilage from the back of the ear, chest wall, or nasal septum represents an additional injury to the patients.

**Figure 11.**
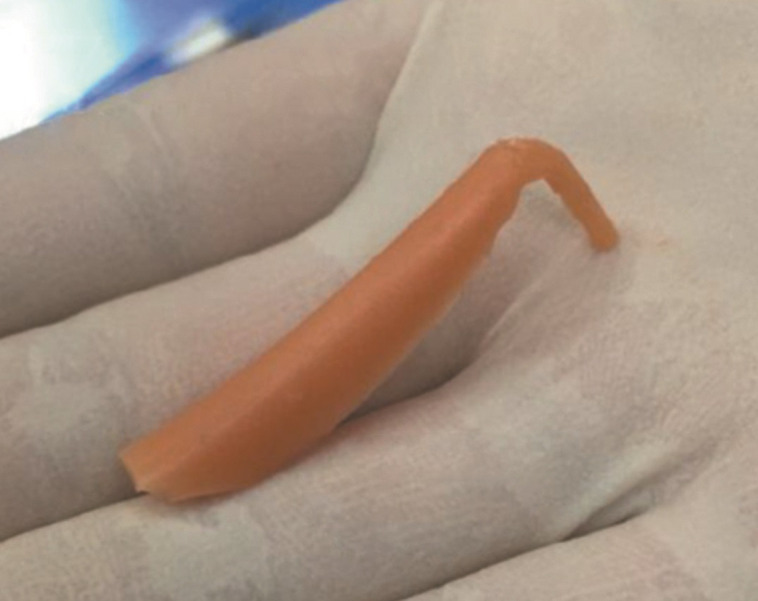
A traditional L-shape silicone implant that has been residing in a human body for 11 years.

Traditional L-shape silicone implants have a long history in rhinoplasty. Silicone has good biocompatibility and antiaging properties. It has been commonly used as a material for implants since World War II.^16^ Figure 11 shows a beige silicone implant that has been in a human body for 11 years.

Silicone nasal implants have multiple advantages. They show minimal change in terms of height and shape and do not deform or deteriorate inside the human body even after a long period.^6^ They are readily available and do not require the use of the patient's autogenous bone, which reduces the pain experienced by the patient at the site of the operation. They are soft and easily tailored, configure, and carve to a desired shape. Silicone implants have virtually no toxicity and antigenicity and can be fused with human tissues. However, traditional silicone implants have greater mobility compared with ePTFE, because silicone cannot adhere to the neighboring tissue.^6^ However, traditional silicone implants still present some complications such as implant deviation, deflection, infection, and extrusion.^8,17^ Hoang et al reported a 9.2% overall complication rate related to silicone nasal implants.^18^ Complications after rhinoplasty are mainly related to the natural effect of gravity, tissue tension around the nasal implant, and external forces. If an implant can solve such postoperative complications, it is considered a very good rhinoplasty material.

To ensure fewer postoperative complications, the Yusha nasal implant includes the following unique changes to its physical structure:

- Convex particle surface: The upper surface of the Yusha nasal implant has staggered columnar protrusions. This rough surface provides diffuse reflection and therefore reduces postoperative translucency. This structure allows fibrous tissue ingrowth, increases the resistance against sliding down, and therefore reduces the possibility of deviation. In addition, the implant is recommended to be carved, as mentioned in the surgical method, to ensure a closer fit to the human nose structure.
- Horizontal and vertical grooves: The Yusha nasal implant has a long vertical groove that is accompanied by 12 to 16 horizontal grooves at the back of the implant, forming an uneven surface that increases the implant's surface area and reinforces resistance against sliding. The grooves also provide the implant a softer touch and reduce its overall weight. This design effectively prevents the implant from deviating from its position after surgery, which also reduces the risk of capsule contraction from high mobility.^19^
- Mantis neck: The novel implant has a narrower neck at the connection point of the nose tip and back. The postsurgical nose tip can therefore move freely as if it is a part of the patient's natural anatomy.
- Hollow nose tip: This novel implant has a hollow nose tip that enables the postsurgical nose tip to feel naturally soft to the touch.

To sum up, the Yusha nasal implant is a novel implant that has antitransparency, antisliding, and anticontraction features to overcome the drawbacks of traditional silicone and ePTFE nasal implants. At the same time, the Yusha nasal implant provides a postoperative outcome that is closer to the natural state.

There are many limitations in this report. First, the number of cases is limited. There were only 114 cases reviewed under this report (46 primary surgery and 68 revisional surgery). All these cases were reviewed by the 2 doctors in my clinic. Although a difference can be noted between the statistics of primary and revisional surgeries, the total number of cases in each group is limited to allow providing a conclusion that using the Yusha implant can eliminate complications in primary surgery. Moreover, I was not able to set a control group to perform further statistical comparisons between the Yusha and other implant types.

## CONCLUSIONS

The idea behind augmentation rhinoplasty is to increase the volume of the nasal structure by using alloplastic materials and integrating those with human tissue. Increasing the surface area of the implant can subsequently expand the contact area between the implant and the human tissue. The Yusha novel nasal implant, with its 4 special structural features, can effectively reduce the postoperative complications of traditional silicone implants, such as sliding, redness, deviation, extrusion, translucency, capsular contracture, and infection. It provides close-to-natural postoperative results with a low risk of complications, thus better satisfying patients’ needs when augmenting noses. It can be concluded that the Yusha nasal implant is a good material for augmentation rhinoplasty.

## Supplemental Material

This article contains supplemental material located online at www.asjopenforum.com.

## Supplementary Material

ojad040_Supplementary_Data
